# Facial Emotion Recognition of 16 Distinct Emotions From Smartphone Videos: Comparative Study of Machine Learning and Human Performance

**DOI:** 10.2196/68942

**Published:** 2025-07-02

**Authors:** Marie Keinert, Simon Pistrosch, Adria Mallol-Ragolta, Björn W Schuller, Matthias Berking

**Affiliations:** 1 Department of Clinical Psychology and Psychotherapy, Friedrich-Alexander-Universität Erlangen-Nürnberg Erlangen Germany; 2 Chair of Health Informatics, Technical University of Munich Munich Germany; 3 Munich Center for Machine Learning Munich Germany; 4 Munich Data Science Institute Munich Germany; 5 Group on Language, Audio, & Music, Imperial College London London United Kingdom

**Keywords:** facial emotion recognition, deep learning, smartphone video, validation, 16 distinct emotions

## Abstract

**Background:**

The development of automatic emotion recognition models from smartphone videos is a crucial step toward the dissemination of psychotherapeutic app interventions that encourage emotional expressions. Existing models focus mainly on the 6 basic emotions while neglecting other therapeutically relevant emotions. To support this research, we introduce the novel Stress Reduction Training Through the Recognition of Emotions Wizard-of-Oz (STREs WoZ) dataset, which contains facial videos of 16 distinct, therapeutically relevant emotions.

**Objective:**

This study aimed to develop deep learning–based automatic facial emotion recognition (FER) models for binary (positive vs negative) and multiclass emotion classification tasks, assess the models’ performance, and validate them by comparing the models with human observers.

**Methods:**

The STREs WoZ dataset contains 14,412 facial videos of 63 individuals displaying the 16 emotions. The selfie-style videos were recorded during a stress reduction training using front-facing smartphone cameras in a nonconstrained laboratory setting. Automatic FER models using both appearance and deep-learned features for binary and multiclass emotion classification were trained on the STREs WoZ dataset. The appearance features were based on the Facial Action Coding System and extracted with OpenFace. The deep-learned features were obtained through a ResNet50 model. For our deep learning models, we used the appearance features, the deep-learned features, and their concatenation as inputs. We used 3 recurrent neural network (RNN)–based architectures: RNN-convolution, RNN-attention, and RNN-average networks. For validation, 3 human observers were also trained in binary and multiclass emotion recognition. A test set of 3018 facial emotion videos of the 16 emotions was completed by both the automatic FER model and human observers. The performance was assessed with unweighted average recall (UAR) and accuracy.

**Results:**

Models using appearance features outperformed those using deep-learned features, as well as models combining both feature types in both tasks, with the attention network using appearance features emerging as the best-performing model. The attention network achieved a UAR of 92.9% in the binary classification task, and accuracy values ranged from 59.0% to 90.0% in the multiclass classification task. Human performance was comparable to that of the automatic FER model in the binary classification task, with a UAR of 91.0%, and superior in the multiclass classification task, with accuracy values ranging from 87.4% to 99.8%.

**Conclusions:**

Future studies are needed to enhance the performance of automatic FER models for practical use in psychotherapeutic apps. Nevertheless, this study represents an important first step toward advancing emotion-focused psychotherapeutic interventions via smartphone apps.

## Introduction

### Background

Emotions are considered essential for human experience and allow individuals to respond adaptively to their environment [1]. Emotions are complex processes, which include behavioral, cognitive, motivational, and physiological components in addition to subjective experience, and most mental disorders are accompanied by emotional disturbances [2]. The most-researched psychotherapy approach, cognitive behavioral therapy, has mainly focused on changing a patient’s behavior and cognition for a long time, but recent developments emphasize the importance of working with a patient’s emotional states to achieve therapeutic changes [3]. The assumed relationship between emotional expression during therapy and therapy outcomes is also supported by meta-analytic evidence [4]. One challenge in modern psychotherapeutic care is the lack of availability of evidence-based treatment, resulting in a large care gap [5]. To bridge this gap and waiting times for psychotherapy, researchers are developing app-based interventions delivered on smartphones that are easily accessible and easy to disseminate [6]. Interventions fostering emotional expressions, however, are difficult to integrate into psychotherapeutic apps. To support the therapeutic process, such apps would need to reliably recognize the emotional expressions shown by patients and be able to provide appropriate feedback. Thus, there is a need for automated emotion recognition that can be integrated into psychotherapeutic apps with comparable performance to humans in detecting patients’ emotional states.

There are 2 main theoretical frameworks to characterize emotional states: the discrete and the dimensional model. The discrete model assumes that emotions can be grouped into distinct and discrete categories. In this regard, the most popular theory is basic emotion theory (BET) [7], which proposes the existence of 6 basic emotions (ie, anger, fear, happiness, sadness, disgust, and surprise) that are universal and characterized by a unique pattern of facial muscle movements. The dimensional model, by contrast, assumes that emotions can be characterized by more or less pronounced expressions on different dimensions. The circumplex model of affect [8], for example, maps emotions on the 2 dimensions of valence and arousal.

One aim of working with emotions in psychotherapy is that patients learn to recognize and differentiate distinct emotional states and make use of their unique features to achieve certain goals [2]. Emotions in such tasks are better conceptualized by discrete models, which is why our work focuses on them. However, the notion of only 6 basic emotions, as proposed by the BET, has been challenged [9], and in psychotherapy, more than these 6 emotions are considered relevant. For example, emotions such as calmness or confidence can be used to support therapeutic change [10]. Therefore, automatic facial emotion recognition (FER) that can reliably recognize more emotional categories that are relevant to therapeutic contexts should be developed.

Currently, there are different approaches to assessing emotional states for automatic emotion recognition. Contact-based methods use physiological sensors to derive emotional states from, for example, heart rate (variability), skin conductance, or muscle tension [11]. Such methods are rather accurate but obtrusive and cannot be integrated into smartphone apps without the help of additional devices. Contactless methods exploit built-in smartphone sensors, such as the camera, the microphone, the GPS, the accelerometer, the gyroscope, the compass, and the light. (refer to the review by Kołakowska et al [12]). Among the contactless, nonobtrusive methods, emotion recognition researchers mainly focused on the study of speech and facial expressions recorded using embedded microphones and cameras [12]. Facial expressions, in particular, convey much information specific to emotional states [13] and are very sensitive to emotional changes [14]. Facial expressions can be encoded with the Facial Action Coding System (FACS) developed by Ekman and Friesen [15]. The FACS allows the description of all facial actions with the help of 44 facial action units (FAUs), which reflect changes in a predefined set of facial muscles. Specific patterns of action units can then be categorized into distinct emotional expressions.

Automatic FER has been explored by several studies, with many approaches relying on FACS and multiple deep learning methods (eg, [16-19]).

Traditional FER systems primarily analyze cropped facial images [20,21], sometimes incorporating additional inputs such as optical flow [21] or phase difference [20] to enhance recognition in videos. Facial features are commonly extracted using convolutional neural networks (CNNs), which are often pretrained on emotion datasets and frozen during training [20,22]. Residual neural network (ResNet) 50, known for its strong performance in facial recognition tasks, has proven to be a robust feature extractor for FER [23,24]. In addition, MobileNetV2 and InceptionV3 have been explored in FER, with a study showing that InceptionV3 outperforms MobileNetV2 in video-based emotion recognition [25].

In addition to deep learning–based feature extraction, handcrafted features remain relevant in FER. Techniques such as Gabor filters, pyramid histogram of orientated gradients, histogram of orientated gradients, and scale-invariant feature transform have been widely used. Among these, FAUs are the most commonly used handcrafted features for FER [26]. The emergence of Automatic Facial Coding has further enhanced FER by combining FAUs with machine learning techniques to facilitate efficient facial expression analysis [27]. Multiple facial features have been successfully processed together in single models to improve FER systems [20-22].

With the advancement of Transformer-based models, Vision Transformers (ViTs) are increasingly being explored for FER because of their ability to leverage long-range dependencies across image patches. Unlike CNNs, ViTs do not rely on convolutional operations but instead partition images into patches and process them using self-attention mechanisms [28]. When fine-tuned on large-scale emotion datasets, ViTs demonstrate promising results in detecting subtle facial expressions [29,30]. The Expression Snippet Transformer enhances facial expression recognition by dividing it into intrasnippet and intersnippet modeling. It uses an attention-augmented feature extractor for detailed intrasnippet encoding and a shuffled snippet order prediction head to capture subtle motion variations [31]. Kim et al [32] used a Swin Transformer architecture within a 3-stream network that integrates visual, temporal, and audio modalities to enhance expression recognition performance.

Beyond facial expressions, multimodal approaches have expanded FER to incorporate additional modalities, such as audio and text. Recent multimodal approaches use CNNs that process multiple inputs, including facial frames, optical flow, and mel spectrograms, to recognize emotions [33]. Another approach uses DistilRoBERTa, a large language model fine-tuned with a combined textual representation of audio and visual features (FAUs). It uses a rule-based system to convert nonverbal cues into text for efficient multimodal emotion recognition [34]. Notably, this study also demonstrated that FACS can still outperform deep learning architectures in certain scenarios [34]. This aligns with other findings indicating that handcrafted features remain relevant. For instance, Gautam and Seeja [35] integrated a histogram of orientated gradients and scale-invariant feature transform with CNNs for FER.

Temporal modeling plays a crucial role in video-based FER, where the relationship between frames must be effectively captured. Recurrent neural networks (RNNs), particularly long short-term memory networks and gated recurrent units (GRUs), are commonly used for this purpose [20,36]. Another approach for modeling temporal relationships is through 3D CNNs [21], which perform convolutions across both the spatial dimensions and the time dimension.

Attention mechanisms have also been important in improving sequential data processing for video FER. The transformer [37], originally designed for language models, has been successfully applied to visual tasks, such as ViTs [28]. The hierarchical contextual attention mechanism (HCAM) uses a 2-level attention mechanism at the word and utterance levels, enabling differential focus on more and less important content [38]. Dutta and Ganapathy [39] used utterance-level hierarchical attention with bidirectional RNNs and self-attention to generate embeddings and then applied a coattention layer to weigh the relevance of these embeddings across audio and text for emotion recognition.

Previous research found the performance of available automatic FER software to be comparable [40,41] or even superior [42] to that of human observers in recognizing emotions in highly standardized pictures of prototypical facial emotional expressions. Regarding FER of dynamic facial expressions, the evidence is less conclusive. For instance, in the study by Krumhuber et al [43], the commercial FER software FACET (iMotions) performed better than human observers in recognizing posed emotional expressions (overall recognition rate of 62.0% from human observers and 69.8% from the software) and equally well in recognizing spontaneous emotional expressions (overall recognition rate of 39.4% from human observers and 45.5% from the software); these results applied to expressions representing the 6 basic emotions. In another study by Krumhuber et al [44], the authors compared the performance of FACET and human observers in recognizing posed and spontaneous emotional expressions from 14 databases and found no performance differences (overall recognition rate of 65.1% from human observers and 65.4% from the software). Recognition rates differed between emotions, with the highest rate found for happiness, followed by disgust, and the lowest for fear. Other studies have also demonstrated the superior performance of human as well as automated FER in recognizing happiness compared to other emotions [45,46]. However, in the study by Dupré et al [45], humans performed better than 8 automatic classifiers in recognizing posed (precision of 72.5% from human observers and 53.9% from the software) and spontaneous emotional expressions. Tcherkassof and Dupré [46] also found human observers’ performance to be superior to automated FER in recognizing self-reports of spontaneous emotional expressions; although in this study, the overall recognition accuracy was low.

### Objectives

Current deep FER models are limited by existing datasets because of their need for large amounts of data. Solutions such as data augmentation, dataset combination, and the creation of new advanced datasets are needed to enhance training effectiveness and reduce overfitting. In addition, further progress in illumination, face poses, and occlusion must be made to enable better generalizable FER [47,48]. This work introduces the novel Stress Reduction Training Through the Recognition of Emotions Wizard-of-Oz (STREs WoZ) dataset for video FER. Unlike many existing datasets focusing on the 6 basic emotions by Ekman [44], the STREs WoZ dataset contains 16 different emotions, enabling more fine-grained emotion classification by distinguishing 4 negative and 12 positive emotions. Each video is assigned to exactly 1 emotion. To the best of our knowledge, with >14,000 videos and 63 participants, it is the largest facial video emotion recognition dataset in an unconstrained laboratory environment [49-53], featuring variations in illumination, face poses, and occlusions. The dataset is specifically tailored for FER exploiting selfie videos, making it particularly suitable for smartphone apps.

Moreover, this study aimed to develop an automatic FER model from the STREs WoZ dataset. Relying on the linguistic content of predefined sentences can limit the model’s applicability in real-world scenarios, where inputs are more varied and unconstrained. Thus, to ensure our emotion recognition models are generalizable and effective in broader contexts, we focused exclusively on the visual modality, enhancing the model’s adaptability to nonconstrained, real-world applications. We approached the problem from two different perspectives: (1) as a binary classification task differentiating between the negative and the positive emotions and (2) as a multiclass classification task targeting the recognition of the 16 fine-grained emotions originally recorded in the dataset. For this purpose, we exploited 2 different types of facial features: appearance and deep-learned features. As a baseline, we modeled the extracted appearance features with a support vector machine (SVM). As the architecture for our deep learning networks, we used 1-directional GRU models with 3 different heads: HCAM, convolution, and average.

Furthermore, this study aimed to validate the newly developed models. To the best of our knowledge, no study so far has evaluated the performance of automatic FER using smartphone selfie videos. Moreover, most studies have only used the 6 basic emotions, although more than those 6 emotions might be relevant for future applications. To fill these research gaps, we evaluated the performance of the models in the test partition of the STREs WoZ dataset and compared it with that of human observers annotating the same test set.

## Methods

### Database

**Figure 1:**
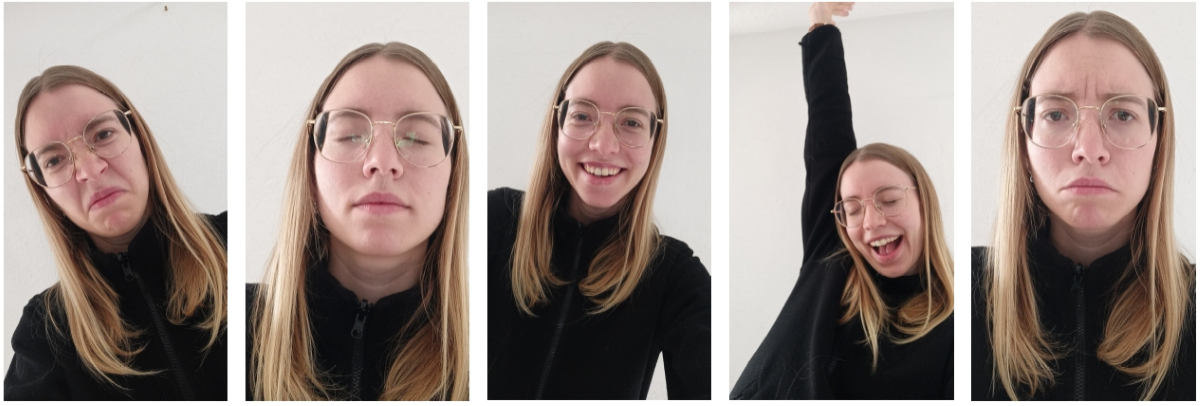
Visualization of facial images representing a subset of the emotions in the dataset. From left to right: disgust, relaxation, happiness, joy, and sadness.

The STREs WoZ dataset is a novel emotion recognition dataset with facial videos in a selfie-style format collected using a smartphone. It contains samples corresponding to 16 different emotions for fine-grained emotion classification, and each video conveys exactly 1 emotion. Of the 16 considered emotions, 4 are negative—anger, disgust, fear, and sadness—and the remaining 12 are positive—confidence, contentment, courage, excitement, gratitude, happiness, joy, love, pride, relaxation, resolve, and tranquility. The emotions were selected by clinical psychologists and experts in psychotherapeutic app development (including the senior author) to represent therapeutically relevant emotions that can be included in psychotherapeutic apps. Figure 1 shows 5 facial frames from the same participant, representing a subset of the emotions considered in the dataset (consent obtained). To the best of our knowledge, this is the largest video-based FER dataset in an unconstrained laboratory environment, with 14,412 videos. The videos were collected in the context of a cognitive restructuring training targeting elevated stress. In the training, participants were required to convey a predefined negative emotion in response to 30 stress-enhancing statements and a positive emotion in response to 30 stress-reducing statements presented to them in random order on a smartphone (for details on the training, refer to the study protocol by Keinert et al [54]). To convey the emotion, participants followed instructions to perform facial expressions and a body movement, and they uttered a predefined German sentence. Participants were free to adjust the specific instructions and personalize the respective emotion display. We fixed the emotion-sentence pairs, and consequently, for each emotion, all participants uttered the same sentence in all the recorded videos. The predefined emotion that participants were supposed to convey served as the videos’ ground truth label.

A total of 63 unique participants took part in our recordings. The sample had a mean age of 22.89 (SD 6.81) years, was 90% (57) university students, and included 12 men and 51 women. Each participant provided 240 videos, with recordings over 4 days with 3 sessions per day. In each session, 1 positive and 1 negative emotion were recorded 10 times. While the positive emotions varied between sessions, the negative emotions remained the same within a day. There were 6 different conditions of the stress reduction training, which differed in the negative emotions to be conveyed. In 4 conditions, participants conveyed the same negative emotion (ie, anger, anxiety, disgust, or sadness) each day. In the remaining 2 conditions, participants conveyed a different negative emotion on each day of the training. This resulted in the number of videos per emotion not being equally distributed in the dataset.

The videos were collected using the front-facing cameras of a Samsung Galaxy S10+ smartphone, which has a 10 MP selfie camera and an 8 MP RGB depth camera. All videos are in portrait format with a frame rate of 30 frames per second. There is usually a white wall in the background of the videos so that the participants’ faces are mainly visible.

Although the videos were recorded in the laboratory, they were recorded under unconstrained conditions. The participants recorded the videos with 1 hand, and they interacted naturally with the smartphone while recording—presenting nonfrontal faces, nonconstant participant-smartphone distances, and free head movements. For these reasons, the resulting videos are not always stable, contain different viewing angles, and the participants’ faces might only be partially visible in some frames. Furthermore, participants were allowed to move inside the recording room.

Three different phases can be identified from the resulting videos: (1) a preparation phase, where participants had already started the recording but were moving the smartphone to the predetermined position for collecting the actual facial video; (2) an emotional phase, where participants convey the desired emotion; and (3) a disengagement phase, characterized by moving the smartphone from the recording position to a more relaxed position to stop the recording.

Data quality is crucial for training deep learning–based models, as it directly impacts their performance. To ensure high-quality inputs for model training, we only included video frames corresponding to the emotional phase. The preparation and disengagement phases were excluded, as they are nonemotional and could introduce noise, potentially degrading the model performance. Focusing solely on the emotional phase reduced the total duration of the dataset, leading to faster processing times and lower memory consumption. To detect the emotional phase, we used an automatic voice activity detector, which recognizes the longest continuous nonsilent segment in each recording as the emotional phase. To ensure that longer-lasting emotions were also captured, 1 second after this segment was included. The accuracy of this procedure was evaluated by analyzing a randomly selected sample of 100 segmented videos. Table 1 provides a summary of the collected data for each emotion. The total duration of the original videos was 17 hours 49 minutes 13 seconds and the total duration of the segmented videos was 7 hours 44 minutes 10 seconds. The disparity in the total duration of each emotion arises from the varying lengths of the utterances associated with each emotion. While some utterances consist of full sentences, others are limited to single words. A list of the utterances is provided in Multimedia Appendix 1.

**Table 1:** Summary of the STREs WoZ^a^ dataset by emotion category.

Emotions	Unique participants, n (%)	Videos (trials), n (%)	Original duration (h:min:s)	Segmented duration (h:min:s)
Anger	31 (49)	1691 (11.73)	4:30:08	1:11:16
Anxiety	29 (46)	1620 (11.24)	5:29:18	1:10:05
Confidence	60 (95)	645 (4.5)	1:31:11	00:13:13
Contentment	59 (94)	635 (4.4)	1:31:40	00:14:13
Courage	60 (95)	645 (4.5)	1:26:30	00:12:47
Disgust	30 (48)	1635 (11.34)	4:48:22	00:58:59
Excitement	60 (95)	645 (4.5)	1:17:27	00:11:07
Gratitude	60 (95)	645 (4.5)	1:28:03	00:10:53
Happiness	60 (95)	645 (4.5)	1:19:31	00:09:52
Joy	62 (98)	669 (4.6)	1:57:33	00:17:53
Love	62 (98)	670 (4.6)	2:02:53	00:23:04
Pride	60 (95)	641 (4.4)	1:36:21	00:17:45
Relaxation	62 (98)	612 (4.2)	2:57:02	00:32:16
Resolve	60 (95)	636 (4.4)	1:29:38	00:13:14
Sadness	31 (49)	1734 (12.03)	6:57:30	1:15:56
Tranquility	60 (95)	645 (4.5)	1:25:58	00:11:30

^a^STREs WoZ: Stress Reduction Training Through the Recognition of Emotions Wizard-of-Oz.

### Development of the Automatic FER Model

#### Facial Features Extraction

##### Overview

The preprocessing stages before feature extraction consisted of face detection and data injection. We used the OpenFace [55] software to detect the faces in each frame of the video. The software produced a cropped image of the detected face with a 224×224 pixels size. In cases where the software failed to detect a face in certain frames, the last extracted facial image was used until a new face was detected in the video. Any gaps occurring at the beginning or end of the video were ignored.

##### Appearance Features

The appearance features investigated correspond to a subset of FAUs, which are based on the FACS and extracted using OpenFace [55]. OpenFace estimates (1) the presence of the FAUs, denoting whether the action unit is visible on the face and (2) their intensity measured on a 5-point scale. OpenFace recognized the FAUs 1, 2, 4, 5, 6, 7, 9, 10, 12, 14, 15, 17, 20, 23, 25, 26, 28, and 45 [55] and predicted the presence and intensity for 17 of these 18 FAUs. For FAU 28 (lip suck), only its presence was predicted. We used all the available features as input for our networks, resulting in a 35D vector for each frame.

##### Deep-Learned Features

The deep-learned features were extracted using a ResNet 50 model [56], which had been pretrained on the VGGFace2 [57] and FER+ datasets [58]. Compared to handcrafted feature extraction techniques, deep features derived from ResNet-50 capture more abstract and high-level representations of facial expressions. In contrast to the FAUs, the resulting 2048D feature vector does not allow us to understand the exact meaning of each individual feature.

#### Data Splitting

Properly training and assessing the performance of the artificial intelligence–based models required splitting the available data into 3 different splits: the first one for training, the second one for validation, and the third one for testing. On the basis of the data partitioning methodology presented in the study by Mallol-Ragolta et al [59], which aims at homogenizing the recognition difficulty among the data partitions, we first followed a leave-one-subject-out cross-validation approach and trained SVMs to measure the unweighted average recall (UAR) performance for each individual participant. The UAR is a measure for determining the performance of a classification model, which is often used for unevenly distributed data. In this study, the UAR was used to evaluate the performance of the models regarding their ability to recognize the correct emotion. Owing to the uneven distribution of the data, this metric assessed whether all emotions were classified effectively rather than only those that occurred more frequently.

Let *A* be a contingency matrix, where *Aij* is the number of instances of a class i classified as *j*. Let *K* be the number of classes. Then, the *UAR* [60] is defined as:

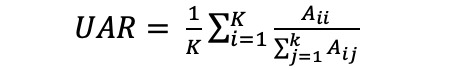



For these initial experiments, the SVMs classified the 16 emotions, and we averaged the extracted FAUs from each video over the time domain to obtain a single, video-level feature representation as input for the SVMs. The UAR scores obtained from each participant varied between 4.4% and 39.8%, with a mean of 20.4% (SD 7.2%). The chance level regarding the UAR for a 16-class classification problem is 6.25%. Next, we sorted the participants in descending order according to the UAR scores obtained. Following a round-robin fashion, the first 3 participants were assigned to the training split, and the fourth and fifth participants to the validation and the test splits, respectively. We repeated this process until all participants were assigned to a data split. The properties of the partitioned dataset are presented in Table 2. The large difference in the SD of the test partition with respect to the training and validation partitions originates from videos with long durations. There were 61, 23, and 2 videos in the training, validation, and test partitions, respectively, that were longer than 10 seconds.

**Table 2:** Summary of the partitioned STREs WoZ^a^ dataset by emotion category.

Partition	Participants, n (%)	Videos (trials), n (%)	Original duration (h:min:s)	Total segmented duration (h:min:s)	Segmented duration (s), mean (SD)
Training	37 (59)	8454 (58.66)	24:02:33	6:39:39	2.83 (1.93)
Validation	13 (21)	2940 (20.40)	9:35:36	2:18:58	2.84 (1.85)
Test	13 (21)	3018 (20.94)	8:02:06	2:15:34	2.70 (1.09)

^a^STREs WoZ: Stress Reduction Training Through the Recognition of Emotions Wizard-of-Oz.

#### Description of Model Architectures

Our baseline model implemented an SVM exploiting the extracted appearance features. The features were preprocessed using standard scaling. The SVM used a radial basis function kernel for classification, with the regularization parameter set to *1* and the kernel’s gamma parameter set to *scale*, which automatically adjusts based on the number of features. The baseline should provide a lower bound for the FER problem, using a nondeep learning approach. We performed grid search optimization on the validation set to determine our hyperparameters.

**Figure 2:**
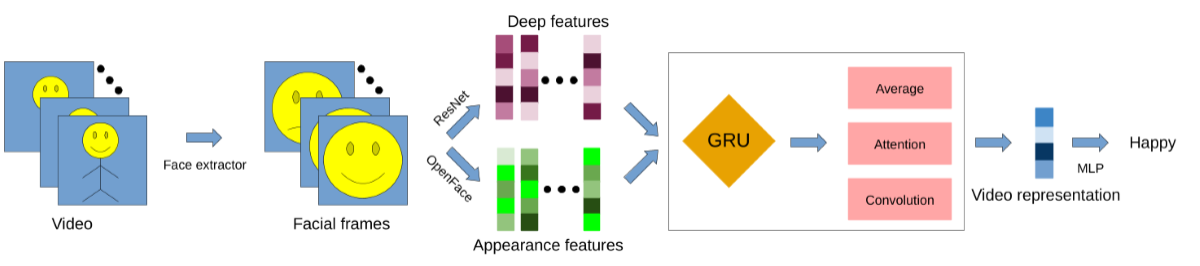
Illustration of the workflow of the deep neural networks. GRU: gated recurrent unit; MLP: multilayer perceptron.

In our deep learning–based experiments, we used the appearance features, the deep-learned features, and the concatenation of both feature types as input to our models. The architecture and parameters of our models were optimized using the validation data. A visualization of the network structures is depicted in Figure 2.

The first block of our networks implemented a 1-directional stacked GRU with 3 hidden layers and a hidden size of 35. The differences among the networks rely on the second block, which combined the outputs of the GRU to obtain a single feature representation to be fed into the multilayer perceptron (MLP) for the final classification. The implementation of the second block of the networks was based on the following methods:

- GRU average—the output of the first block was reduced to a video-level feature representation using a simple average. This feature representation was passed to the MLP.
- GRU HCAM—an HCAM was implemented to obtain a video-level feature representation. The features of each frame were treated analogously to the sentence embeddings in a document using the attention mechanism presented by Yang et al [38].
- GRU convolution—this block implemented a single 1D convolutional layer with a kernel size of 3, a stride of 1, and the same padding, followed by an adaptive average pooling with a 1D output over the sequence length for each feature. The convolutional layer applied the kernel to the same feature in multiple consecutive frames, considering past and future information to determine the salient representation of the current frame.

The third block was composed of an MLP, which implemented 2 hidden layers, each containing 24 hidden units and rectified linear unit as the activation function. The final activation function of this block was a softmax function. For a fair comparison among the models, we trained all our networks with Adam as the optimizer, a learning rate of 0.001, and a batch size of 64. We also implemented an early stopping mechanism with a patience of 30 epochs to minimize the risk of overfitting.

The models were trained on a GTX Titan X, equipped with 12 GB of GDDR5 memory. This setup provided sufficient resources for the training process, allowing us to handle the computational demands of the models. Training times varied depending on the architecture, with more complex models requiring longer processing periods because of the increased number of parameters and computational operations. The batch size was adjusted to optimize performance while ensuring efficient memory use. The SVM and deep learning models were implemented using Python (Python Software Foundation) and the following libraries: *scikit-learn* for the SVM implementation and *PyTorch* for the deep learning models.

### Performance Evaluation of the Automatic FER Model

#### Description of the Test Set

The test set used for the validation of the newly developed models was taken from the STREs WoZ dataset and comprised 13 individuals displaying 1 of the 16 emotions. All individuals were women, with a mean age of 20.92 (SD 2.06; range 18-24) years, White, and—except for 1—reported elevated subjective stress levels. The test set included 3018 videos. For the validation with human observers, they were not only shown the emotional phase but the entire videos. We opted for this approach to allow a realistic comparison between automated and human assessment, as preprocessing of the data, including extraction of the relevant phase, is an integral part of the automated processing pipeline. As a result, the average duration of the videos shown to human observers was 8.5 (SD 4.79) seconds. The dataset included the following emotions: anger (316/3018, 10.47%), anxiety (534/3018, 17.69%), disgust (77/3018, 2.55%), sadness (406/3018, 13.45%), confidence (145/3018, 4.8%), contentment (135/3018, 4.47%), courage (144/3018, 4.77%), excitement (135/3018, 4.47%), gratitude (144/3018, 4.77%), happiness (135/3018, 4.47%), joy (144/3018, 4.77%), love (144/3018, 4.77%), pride (145/3018, 4.8%), relaxation (145/3018, 4.8%), resolve (135/3018, 4.47%), and tranquility (134/3018, 4.44%). As we aimed to focus exclusively on the visual modality, the videos were muted for all purposes of this study.

#### Human Observers

A total of 3 psychology students (2 women, 1 man; mean age 28.33 (SD 2.08) years) participated in the study as human observers. Before completing the test set, they underwent a training in which they discussed the typical features of each target emotion (refer to Multimedia Appendix 1 for the features). Subsequently, they viewed sample video clips of an actor displaying each emotion, followed by classification practice. For this purpose, we used a training set of 144 videos from the STREs WoZ dataset that were not part of the test set. The training set was drawn from 3 randomly selected participants and comprised 3 videos per emotion per participant. Human observers were aware of the respective emotions displayed in the videos, allowing them to study and familiarize themselves with the defining features of each emotional expression by watching each video carefully.

To prepare the test set for human annotation, video file names were replaced with random numbers generated using a web-based tool [61], preventing any bias based on the file name or sequence. The human observers were instructed to watch all 3018 video clips carefully, with adequate rest periods to maintain focus. Human observers’ emotion recognition was assessed using a forced-choice task, in which they were required to categorize each video based on the valence of the emotion (positive vs negative) and select the specific emotion displayed from a list of 16: anxiety, anger, disgust, sadness, joy, relaxation, love, excitement, tranquility, gratitude, happiness, resolve, contentedness, courage, confidence, and pride.

#### Data Analysis

For the performance evaluation and comparison with human observers, we selected the model with the best overall performance in the validation data. The performance of the deep learning model and the human observers in both binary (positive vs negative) and multiclass (distinguishing the 16 emotions) classification tasks was evaluated using accuracy 
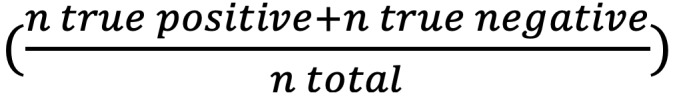
 and UAR. For human observers, we calculated the mean and SD of the accuracy, as well as the bootstrapped CIs with 1000 replicates. In addition, we used a 1-sided binomial test to determine whether the accuracy exceeds chance with a significance level of α=.05. To assess interrater reliability, including agreement between human observers and between human and model inference, Fleiss κ and Cohen κ were calculated. We followed standard heuristics, interpreting κ values as indicating slight (κ≤.2), fair (0.2<κ≤0.4), moderate (0.4<κ≤0.6), substantial (0.6<κ≤0.8), or almost perfect (0.8<κ) agreement [62]. All analyses were conducted using RStudio (Posit, PBC, version 4.4.0) [63]. Performance metrics were calculated using the confusionMatrix function from the *caret* package [64], and the interrater reliability was computed using the *irr* package [65].

### Ethical Considerations

This study adhered to the ethical principles outlined in the Declaration of Helsinki and received approval from the Ethics Committee of the German Psychological Society (BerkingMatthias2020-09-10AM). Participants were informed by the study staff about the study procedures and the inherent impossibility to fully anonymize video data. Subsequently, they provided written informed consent for the collection and analysis of their recordings. To ensure data security, all video files were stored under a unique personal code for each participant. Given the limitations on anonymization, the data were stored on a password-protected hard drive that was securely locked at all times.

As compensation, participants were given the opportunity to enter a €500 (US $ 567.52) lottery. Psychology students could alternatively receive course credits.

## Results

### Development of the Automatic FER Model

As baseline, we trained an SVM on the mean appearance features of the videos. We obtained a UAR of 85.1% for the binary classification on the validation partition and a UAR of 89.3% on the test partition. The SVM achieved a UAR of 29.2% for the multiclass classification on the validation partition and 33.5% on the test partition. All our GRU networks with appearance features performed better than the SVM baseline. The deep features and feature concatenation did not lead to performance improvements, as their effectiveness largely depended on the specific architecture in which they were integrated. The attention head seemed to be the most suitable for all features, as it obtained their best performance. Table 3 shows the results of the various approaches for the binary classification task. The attention networks obtained the best performance on both the validation and the test sets, outperforming the other heads in nearly every experiment, except for the GRU Convolution approach in the test set.

**Table 3:** Results of the binary emotion classification task reported using unweighted average recall.

		GRU^a^ average, %	GRU attention, %	GRU convolution, %	SVM^b^ baseline, %
**Validation**					
	AFs^c^	88.2	89.3	88.9	85.1
	DFs^d^	87.6	89.1	87.7	N/A^e^
	Both	88.9	*90.7* ^f^	86.2	N/A
**Test**					
	AFs	90.5	*92.9* ^f^	92.7	89.3
	DFs	86.2	90.8	85.0	N/A
	Both	88.1	90.4	92.6	N/A

^a^GRU: gated recurrent unit.

^b^SVM: support vector machine.

^c^AF: appearance feature.

^d^DF: deep feature.

^e^N/A: not applicable.

^f^Italicization indicates the best performance for the validation and test partitions.

In the multiclass classification task (Table 4), the attention head clearly enhanced the exploration of feature potential across both sets. While the convolution head improved the model performance on the validation set, its results on the test set were mixed, sometimes performing worse and other times better than the average approach. When analyzing the different features, the appearance and deep features performed almost equivalently on the validation set for the binary task. However, on the test set, the appearance features outperformed the deep features. The concatenation of both features showed a small performance gain on the validation set, but in some cases, they led to a decline in performance on the test set.

**Table 4:** Results of the multiclass emotion classification task reported using unweighted average recall.

			GRU^a^ average, %		GRU attention, %		GRU convolution		SVM^b^ baseline, %
**Validation**									
	AFs^c^	38.6		*44.6* ^d^		41.3		29.2	
	DFs^e^	22.4		35.3		23.7		N/A^f^	
	Both	19.2		29.2		23.6		N/A	
**Test**									
	AFs	47.3		*52.9* ^d^		44.8		33.5	
	DFs	30.1		41.7		31.2		N/A	
	Both	30.9		42.0		30.4		N/A	

^a^GRU: gated recurrent unit.

^b^SVM: support vector machine.

^c^AF: appearance feature.

^d^Italicization indicates the best performance for the validation and test partitions.

^e^DF: deep feature.

^f^N/A: not applicable.

For the multiclass classification task (Table 4), the concatenated features offered minimal improvement over the baseline. Only the GRU attention approach on the test set outperformed the baseline. The deep feature networks with the attention head scored higher UAR scores than the baseline in both validation and test sets. The appearance feature networks achieved the best results in both validation and test sets.

### Performance Evaluation of the Automatic FER Model

For the performance evaluation, we selected the best overall model (ie, the model with attention head and appearance features), as it performed the strongest on the validation data.

#### Binary Classification

The attention network with appearance features achieved an overall accuracy of 92.2% (95% CI 91.2-93.2) and a UAR of 92.9% (Table 3) for the binary classification, which was significantly greater than chance (*P*<.001) with Cohen κ=0.84. The human observers achieved a mean accuracy of 91.1% (SD 0; 95% CI 90.9-91.4) and a UAR of 91.0% for the binary classification, which was also significantly greater than chance (*P*<.001). The overall agreement among human observers was high (Fleiss κ=0.987), and the agreement on correct classifications was similarly high (Fleiss κ=0.961). The mean agreement between the attention network and the human observers’ classification was substantial, with mean Cohen κ=0.70 (SD 0).

#### Multiclass Classification

**Figure 3:**
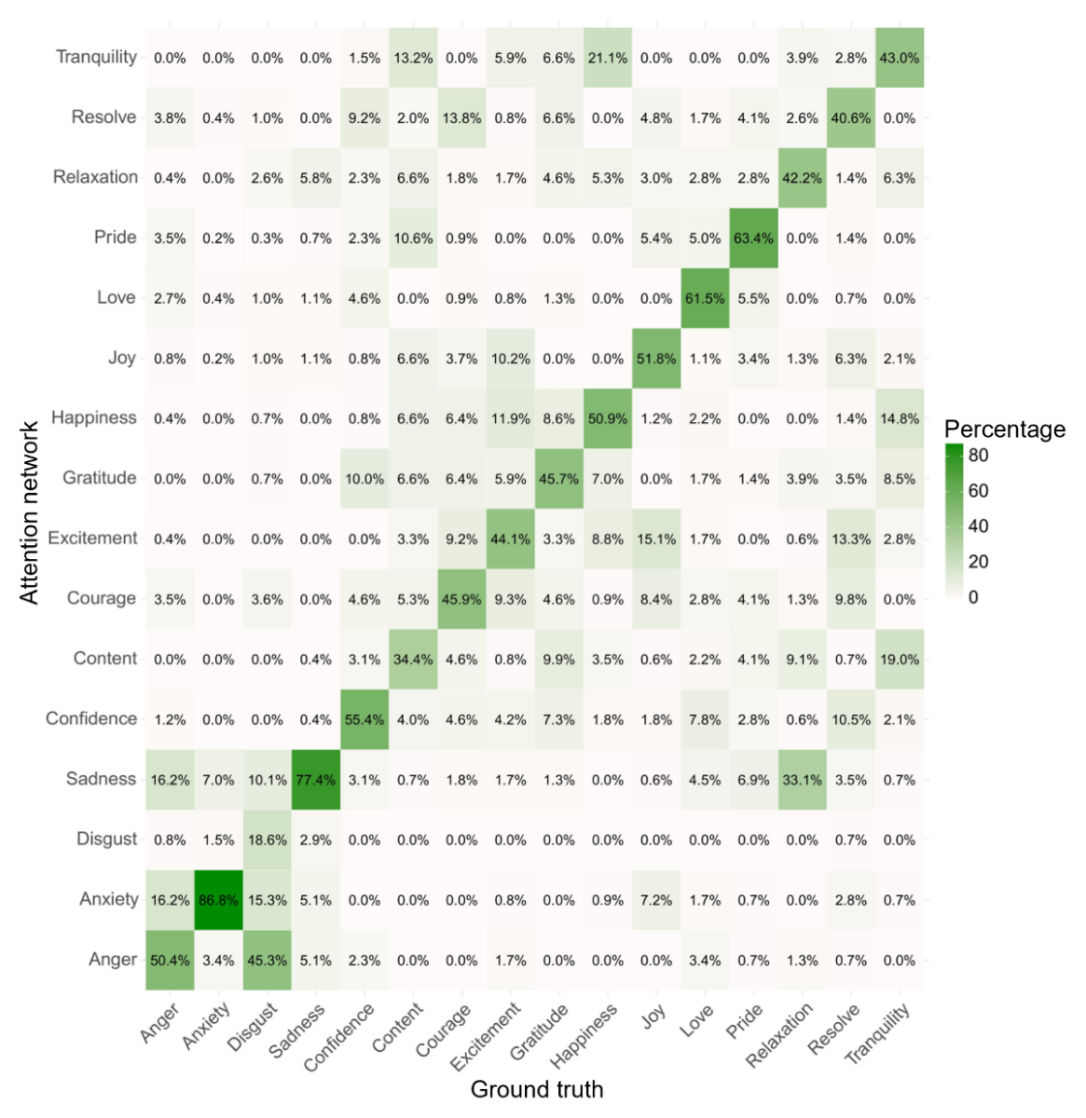
Confusion matrix of the attention network’s multiclass classification.

**Figure 4:**
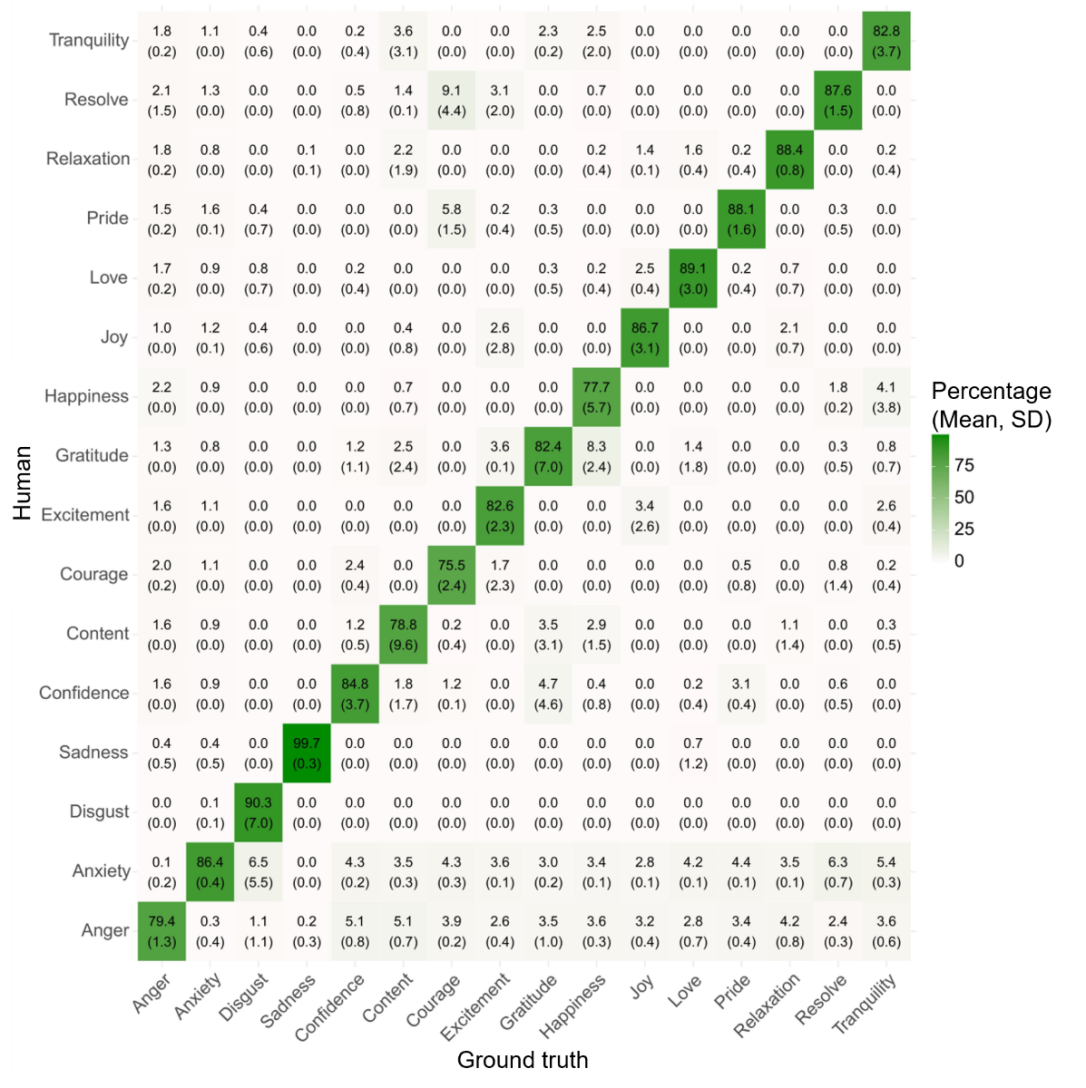
Confusion matrix of the human observers’ multiclass classification.

The attention network’s multiclass classification achieved an overall accuracy of 54.2% (95% CI 52.4-56) and a UAR of 52.9% (Table 4), which was significantly greater than chance (*P*<.001) with Cohen κ=0.50. UARs of the fine-grained emotions ranged between 59.0% for disgust and 90.0% for anxiety. The human observers achieved a mean accuracy of 85.9 (SD 0.02; 95% CI 84.1-87.5]), also significantly greater than chance (*P*<.001). The human observers’ overall agreement (Fleiss κ=0.927) and the agreement of correct classifications (Fleiss κ=0.758) was substantial to high. Accuracies of the fine-grained emotions ranged between 87.4% for courage and 99.8% for sadness. The confusion matrices for single emotion classifications by both the attention network and the human observers are displayed in Figures 3 and 4, respectively. The mean agreement between the attention network’s and the human observers’ classification was moderate, with mean Cohen κ=0.45 (SD 0). The balanced accuracies for both the attention network and the human observers are displayed in Table 5. For the individual performance metrics, such as accuracy, sensitivity, specificity, precision, and *F*_1_-scores, refer to Tables S1 and S2 in Multimedia Appendix 2.

**Table 5:** Accuracy of the attention network’s and the human observers’ classification.

Emotion	Attention network, %	Human observers, %
Anger	71.9	88.5
Anxiety	90.9	91.7
Disgust	59	95.1
Sadness	85.2	99.8
Confidence	76.4	91.9
Contentment	65.8	89
Courage	71.3	87.4
Excitement	70.6	91
Gratitude	71.5	90.6
Happiness	74.1	88.5
Joy	74.9	93
Love	80.1	94.2
Pride	80.8	93.7
Relaxation	69.7	93.9
Resolve	68.9	93.2
Tranquility	70.2	91

## Discussion

### Principal Findings

This study focused on developing and evaluating an automatic FER model for the classification of 16 discrete, therapeutically relevant emotions from the STREs WoZ dataset. The dataset contains 14,412 selfie videos of posed emotions collected via a smartphone app in the context of a stress reduction training. The FER models performed a binary classification task (ie, distinguishing positive and negative emotions) and a multiclass classification task (ie, distinguishing the 16 discrete emotions).

Regarding the development of automatic FER models, we exploited both appearance and deep features. Notably, the appearance features outperformed the deep features. This was an unexpected result, given that deep features are typically more complex and can, therefore, capture more salient information. A possible explanation is that the deep features were not fine-tuned on the faces in this dataset. When both features were concatenated, the performance even declined in some cases, potentially because of the dominance of the deep features, which may have limited the capabilities of the appearance features. The results also indicate that a head with HCAM was more effective in creating feature representations than the averaging or the convolutional approaches.

A notable finding was the large performance gain on the test set compared to the validation set. This can likely be attributed to the increased availability of training data, as both the training and validation data were used for training before evaluating the test data. This approach may have facilitated improved differentiation and generalization of emotions. Consequently, extending the STREs WoZ dataset with more participants could be beneficial for future research, as it may enhance the generalization of emotions and further improve model performances.

Regarding the performance evaluation, the attention network’s accuracy in the binary classification task was high (92.2%) and comparable to human observers’ performance, who had a mean accuracy of 91.1%. In terms of classifying the 16 fine-grained emotions, the attention network’s accuracy ranged between moderate (59.0% for disgust) and almost perfect (90.0% for anxiety), whereas human observers achieved a mean accuracy of at least 87.4%. In this study, the automatic classifier’s recognition accuracy for posed dynamic facial emotional expressions ranged from lower [43,44] to comparable [45] compared to other tools reported in the literature, while human observers demonstrated superior overall accuracy. However, those studies only investigated automatic FER of the 6 basic emotions. To the best of our knowledge, this study is the first to investigate automatic FER of 16 distinct emotion categories. This might explain the inferior performance of the automatic classifier in our study, as differences between these emotions are more subtle than between the 6 basic emotions and, therefore, more difficult to distinguish.

In addition, although emotional expressions in the STREs WoZ dataset were posed, participants were free to adjust the specific instructions and personalize their emotional displays. Consequently, the emotional expressions in our dataset are probably less prototypical than in other datasets of posed expressions, making FER more challenging. This might explain why human observers were superior in recognizing the fine-grained emotions, as they might be better at attributing subtle changes to the correct emotion. However, it is surprising that human observers in our study outperformed those in previous studies. It is possible that they were able to recognize the predefined sentences that participants uttered when conveying the respective emotion and based their categorization mainly on that rather than on the facial features, which might explain our finding.

Our work advances theoretical development in the field by, to the best of our knowledge, being the first to apply automatic FER to 16 distinct, therapeutically relevant emotions. Previous studies have primarily focused on the 6 basic emotions proposed by BET [7], which do not fully capture the complexity of human emotional expression and the variety of emotional states that are relevant in psychotherapeutic contexts. The confusion matrix results from our study, particularly the relatively low confusion among most positive emotions, suggest that these emotions indeed contain distinct elements, supporting the validity of distinguishing >6 emotions. This approach more accurately reflects the diversity of emotional experiences and provides a stronger foundation for future research in emotion recognition.

Regarding the clinical relevance of our findings, developing automatic FER models for smartphone selfie videos represents a significant step toward integrating emotion recognition into psychotherapeutic app interventions. However, it remains questionable whether the performance achieved in this study is sufficiently high to ensure the effective functioning of such apps. While the 92.2% accuracy in the binary classification task was comparable to human performance and likely adequate, the 59.0% to 90.0% accuracy in multiclass classification—though significantly above chance—was far inferior overall to that of human observers. Using a model that correctly classifies just over half of the cases for an app designed to provide feedback and respond based on accurate classification is not feasible. The high rate of incorrect classifications would likely cause user irritation and frustration. However, when working with patients’ emotional states in psychotherapeutic app interventions, recognizing different emotion categories beyond merely differentiating positive and negative is essential. Therefore, to make the integration of automatic FER into such apps feasible, improvements in the model performance are necessary. This could likely be achieved by extending the STREs WoZ dataset to include more participants. Our study demonstrated that increased data can enhance performance, as evidenced by the performance gain in the test set compared to the validation test. Another approach might involve using multimodal strategies to boost accuracy, such as incorporating eye movement or gaze information into the models. Audio could also be used for better emotion recognition. However, caution is required when training models using speech, as there is a risk that the model may learn artificial patterns for emotion recognition. This concern arises from the fact that, in the dataset used in this study, each emotion is represented by only a single distinct utterance.

### Limitations

This research has several limitations to be considered. First, our current model relies on older handcrafted features, such as FAUs, or features extracted from older architectures, such as ResNet-50, which, while useful for establishing a benchmark, are not as effective as newer, more powerful models. Future work should involve exploring more recent and advanced models, such as ViTs or Swin Transformers, which have demonstrated superior performance in many vision tasks, including emotion recognition. These models, though computationally demanding, are likely to offer significant improvements in multiclass emotion recognition and may be crucial for the successful integration of FER into psychotherapeutic app interventions.

Second, the posed nature of the emotions in our dataset limits the use of our model for real-world applications in naturalistic settings. In-the-wild emotions can differ in appearance and thus cannot be recognized well with systems trained on posed emotions. However, by recording the videos as selfie videos, the STREs WoZ dataset shows real-world properties for perspectives, recording angles, head movements, and shakiness, and thus takes a first step toward better generalizability to real-world settings. Moreover, the recognition of posed emotion displays might be relevant for certain psychotherapeutic apps where patients are instructed to adopt certain poses or facial expressions to evoke emotional states based on embodiment theories [66]. Third, a further limitation of generalizability in this study is the lack of cross-cultural validation of the 16 emotion categories. To make our results applicable to other cultures, this will be a crucial step for future studies. Fourth, our sample was homogeneous regarding age, gender, and ethnicity. Faces change with age; therefore, recognizing emotions in older people becomes much more challenging. Older people are less expressive and have less elaborated emotion schemas [67]. Moreover, typical facial features differ between genders and ethnicities. Therefore, there is a risk of bias in the models developed with our dataset. In a follow-up study, we are already working on collecting data from a more diverse participant pool (ie, regarding age, gender, ethnicity, and clinical variables) to increase generalizability. In addition, future research could leverage the potential of artificial intelligence to generate more diverse datasets for the development of bias-free FER models. Fifth, the different input conditions of automatic and human FER in our study may also have introduced bias in the performance comparisons. Human observers viewed the entire videos, including the preparation and disengagement phases, whereas the automatic classification was based solely on the emotional phase. Although we opted for this approach to reflect a realistic comparison between automatic and human FER, human observers may have had access to additional contextual information that aided in recognizing the correct emotion. Finally, because the emotional displays in the videos were accompanied by the utterance of a predefined sentence that varied little between participants, human observers likely guessed the sentence through lip-reading and based their categorization on that, making the task easier and potentially boosting their performance. In addition, the predefined sentences may have created artificial patterns in the data, introducing biases in emotion recognition. Future studies should enhance the comparability of human and automatic FER by minimizing the possibility of lip-reading and using spontaneous utterances.

### Conclusions

Despite these limitations, this research makes a valuable contribution by developing models for automatic FER of 16 distinct, therapeutically relevant emotions from smartphone videos. Consequently, it represents an important first step toward the development and integration of automatic FER into psychotherapeutic apps, paving the way for more personalized and effective digital mental health interventions.

## Appendix

Multimedia Appendix 1 Typical features of the 16 target emotions.

Multimedia Appendix 2 Performance indicators of the attention network and human observers.

## Abbreviations

BET: basic emotion theory
CNN: convolutional neural network
FACS: Facial Action Coding System
FAU: facial action unit
FER: facial emotion recognitionGRU: gated recurrent unit
HCAM: hierarchical contextual attention mechanism
MLP: multilayer perceptron
ResNet: residual neural network
RNN: recurrent neural network
STREs WoZ: Stress Reduction Training Through the Recognition of Emotions Wizard-of-Oz
SVM: support vector machine
UAR: unweighted average recall
ViT: Vision Transformer

## Data Availability

The video dataset analyzed during this study is not publicly available because of data protection reasons. The code for the different models as well as instructions on how to run them will be shared via GitHub [68].
