# Supplementary material for: Facial Emotion Recognition of 16 Distinct Emotions From Smartphone Videos: Comparative Study of Machine Learning and Human Performance
Source: J Med Internet Res. 2025 Jul 2;27:e68942. doi: 10.2196/68942 (PMC12268218; doi:10.2196/68942)
Supplement: Multimedia Appendix 1 [file jmir_v27i1e68942_app1.pdf]

Table S1. Typical features of the 16 target emotions.

	**Facial expression**	**Body movement**	**Utterance**
**Anger**	angry facial expression	tense body, clench a fist, hit the table	"Such a stupid belief, I won't listen to its advice!"
**Anxiety**	anxious facial expression	Withdraw body quickly	"It is dangerous to think like that, I will protect myself from that!"
**Disgust**	disgusted facial expression	Avert face	"Ugh, this is disgusting, I will stay away from that!"
**Sadness**	Sad facial expression	Lower head and let it hang down	"I will let go of this belief!"
**Confidence**	smile	nod	"That's how it is done!"
**Content**	Smile	nod	"That's good."
**Courage**	Look ahead	Clench a fist, nod	"I can do that!"
**Excitement**	Smile	Raise arm in the air	"Yeah!"
**Gratitude**	Smile	Friendly nod	"Thanks a lot!"
**Happiness**	Smile	Nod	"Yes."
**Joy**	Smile	Joyful leap	"Yay!"
**Love**	Nod	Take a hand to the heart	"Such a beautiful belief!"
**Pride**	Smile to oneself	Stick out chest, pat shoulder with the hand	"You've done a great job!"
**Relaxation**	Close eyes	Relax muscles, breathe deeply	"That's nice."
**Resolve**	Nod	Approach determinedly	"I'm going to tackle it!"
**Tranquility**	Nod	Thumb up	"Exactly."
