# Supplementary material for: Facial Emotion Recognition of 16 Distinct Emotions From Smartphone Videos: Comparative Study of Machine Learning and Human Performance
Source: J Med Internet Res. 2025 Jul 2;27:e68942. doi: 10.2196/68942 (PMC12268218; doi:10.2196/68942)
Supplement: Multimedia Appendix 2 [file jmir_v27i1e68942_app2.pdf]

Table S2. Performance indicators of the attention network. UAR: Unweighted Average Recall.

	UAR	Sensitivity	Specificity	Precision	F1
**Binary classification**	92.9 %	.92	.93	.91	.91
**Multi-class classification**					
**Anger**	71.0 %	.50	.93	.42	.46
**Anxiety**	90.9 %	.87	.95	.76	.81
**Disgust**	59.0 %	.19	.99	.76	.30
**Sadness**	85.2 %	.77	.93	.52	.62
**Confidence**	76.4 %	.55	.97	.50	.52
**Content**	65.8 %	.34	.97	.39	.36
**Courage**	71.3 %	.45	.97	.35	.40
**Excitement**	70.6 %	.44	.97	.39	.41
**Gratitude**	71.5 %	.46	.97	.48	.47
**Happiness**	74.1 %	.51	.97	.43	.47
**Joy**	74.9 %	.52	.98	.60	.56
**Love**	80.1 %	.61	.99	.76	.68
**Pride**	80.8 %	.63	.98	.63	.63
**Relaxation**	69.7 %	.42	.97	.45	.43
**Resolve**	68.9 %	.41	.97	.43	.42
**Tranquility**	70.2 %	.43	.97	.46	.44

Table S3. Performance indicators of human observers. UAR: Unweighted Average Recall.

	**UAR** *M* (*SD*)	**Sensitivity** *M* (*SD*)	**Specificity** *M* (*SD*)	**Precision** *M* (*SD*)	**F1** *M* (*SD*)
**Binary classification**	91.0 % (0.2 %)	.89 (.00)	.92 (.00)	.90 (.00)	.90 (.00)
**Multi-class classification**					
**Anger**	88.5 % (0.5 %)	.79 (.01)	.98 (.00)	.80 (.01)	.79 (.01)
**Anxiety**	91.7 % (0.2 %)	.86 (.00)	.97 (.00)	.86 (.01)	.86 (.01)
**Disgust**	95.1 % (2.9 %)	.90 (.06)	.99 (.00)	.99 (.01)	.94 (.03)
**Sadness**	99.8 % (0.2 %)	.99 (.00)	.99 (.00)	.99 (.01)	.99 (.01)
**Confidence**	91.9 % (1.6 %)	.85 (.03)	.99 (.00)	.81 (.05)	.83 (.04)
**Content**	89.0 % (4.0 %)	.79 (.08)	.99 (.00)	.83 (.03)	.81 (.05)
**Courage**	87.4 % (0.9 %)	.76 (.02)	.99 (.00)	.86 (.03)	.80 (.01)
**Excitement**	91.0 % (1.0 %)	.83 (.02)	.99 (.00)	.86 (.02)	.84 (.02)
**Gratitude**	90.6 % (3.0 %)	.82 (.06)	.99 (.00)	.76 (.07)	.79 (.06)
**Happiness**	88.5 % (2.4 %)	.78 (.05)	.99 (.00)	.85 (.03)	.81 (.04)
**Joy**	93.0 % (1.3 %)	.87 (.03)	.99 (.00)	.88 (.02)	.87 (.02)
**Love**	94.2 % (1.2 %)	.89 (.02)	.99 (.00)	.88 (.01)	.89 (.02)
**Pride**	93.7 % (1.0 %)	.88 (.01)	.99 (.00)	.84 (.01)	.86 (.01)
**Relaxation**	93.9 % (0.3 %)	.88 (.01)	.99 (.00)	.87 (.03)	.88 (.01)
**Resolve**	93.2 % (0.6 %)	.88 (.01)	.99 (.00)	.73 (.06)	.79 (.03)
**Tranquility**	91.0 % (1.6 %)	.83 (.03)	.99 (.00)	.81 (.05)	.82 (.04)
